# 

**Published:** 1877-09

**Authors:** 


ESTABLISHED IN 1855.

VOLUME XV SEPTEMBER—1877 NUMBER 6.

### ATLANTA
### Medical and Surgical Journal.

MONTHLY: THREE DOLLARS PER ANNUM, IN ADVANCE.

EDITOR:

J. G. WESTMORELAND, M.D.

*Professor Materia Medica in Atlanta Medical College.*

ASSOCIATE EDITORS:

R. W. WESTMORELAND, M.D.,

*Assistant Demonstrator and Prosector of Anatomy in Atlanta Medical College.*

SHIRLEY BRAGG, M.D.,

H. H. DICKSON, PUBLISHER.

*PAX ET SCIENTIA, SED VERITAS SINE TIMORE.*

ATLANTA, GEORGIA:

*H. H. DICKSON, BOOK AND JOB PRINTER AND BOOKBINDER.*

